# Crystal structure of (*E*)-3-[(2,6-di­methyl­phen­yl)diazen­yl]-7-methyl-1*H*-indazole

**DOI:** 10.1107/S2056989018012483

**Published:** 2018-09-14

**Authors:** Shiomi Yagi, Tomoyuki Haraguchi, Takashiro Akitsu

**Affiliations:** a Department of Chemistry, Faculty of Science, Tokyo University of Science, 1-3 Kagurazaka, Shinjuku-ku, Tokyo 162-8601, Japan

**Keywords:** crystal structure, photochromism, azo benzene, pyrazole, indazole, chiral crystallization

## Abstract

The title azo compound, C_16_H_16_N_4_, was synthesized from 2,6-di­methyl­aniline. The diazenyl group adopts a *trans* (*E*) conformation, with an N=N bond length of 1.265 (4) Å. The pyrazole ring is approximately planar. In the crystal, zigzag chains along the *b*-axis direction with a *C*(3) is graph-set motif are formed by N—H⋯N hydrogen bonds involving the pyrazole moiety.

## Abstract 2

The title compound is composed of a benzene ring linked to an indazole unit by an N=N bond and has high planarity. The diazenyl group adopts a *trans* (*E*) conformation.

## Chemical context   

Azo­benzene derivatives are known to be photochromic com­pounds and numerous studies have been reported (Aritake *et al.*, 2011 ▸; Bobrovsky *et al.*, 2016 ▸; Li *et al.*, 2017 ▸). As an example of this, our group has reported the crystal structures of several azo­benzene derivatives (Moriwaki & Akitsu, 2015 ▸; Moriwaki *et al.*, 2017 ▸).
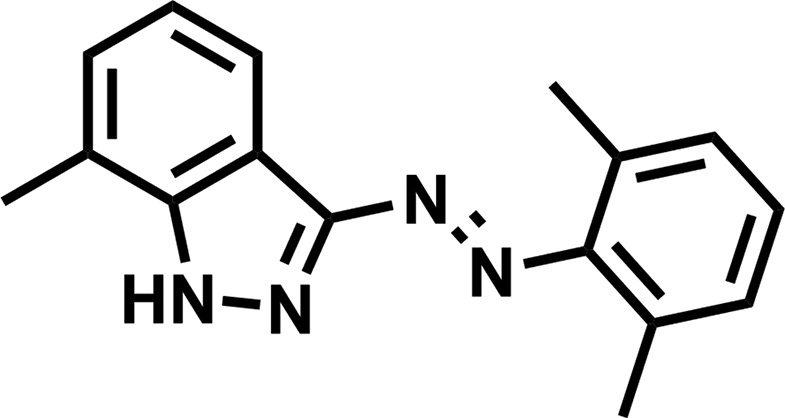


Pyrazole is an aromatic compound comprising a five-membered ring with two adjacent N atoms. Pyrazole derivatives are biologically active and have attracted attention for the synthesis of new medicinal products (Ansari *et al.*, 2017 ▸).

**Figure 1:**
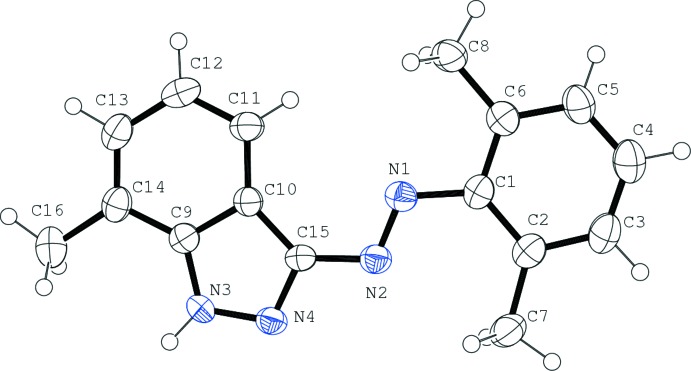
The structure of the title compound shown with 50% probability displacement ellipsoids.

Here we report the crystal structure of (*E*)-3-[(2,6-di­methyl­phen­yl)diazen­yl]-7-methyl-1*H*-indazole, which has an azo­benzene moiety and a pyrazole moiety (Fig. 1 ▸).

## Structural commentary   

The mol­ecular structure of the title compound is composed of a benzene ring linked to an indazole unit by an N=N bond. In the azo­benzene moiety, the azo N=N double bond adopts an *E* configuration, with an N=N bond length of 1.265 (4) Å and a corresponding C9—N3—N4—C15 torsion angle of 0.7 (4)°.

The mol­ecule is practically flat with a maximum deviation of 0.142 (5) Å (for atom C7) from the mean plane passing through the non-H atoms. The pyrazole ring (N3/N4/C15/C10/C9) is approximately planar with an r.m.s. deviation of 0.0026 Å. The C—C bond lengths of the pyrazole ring are 1.404 (6) and 1.428 (5) Å, the C—N bond lengths are 1.322 (5) and 1.359 (5) Å and the N—N bond length is 1.351 (4) Å, in good agreement with values reported previously for 7-methyl-1*H*-indazole [1.400 (4), 1.422 (4), 1.320 (4), 1.366 (3) and 1.356 (3) Å, respectively; Foces-Foces, 2005 ▸]

## Supra­molecular features   

**Figure 2:**
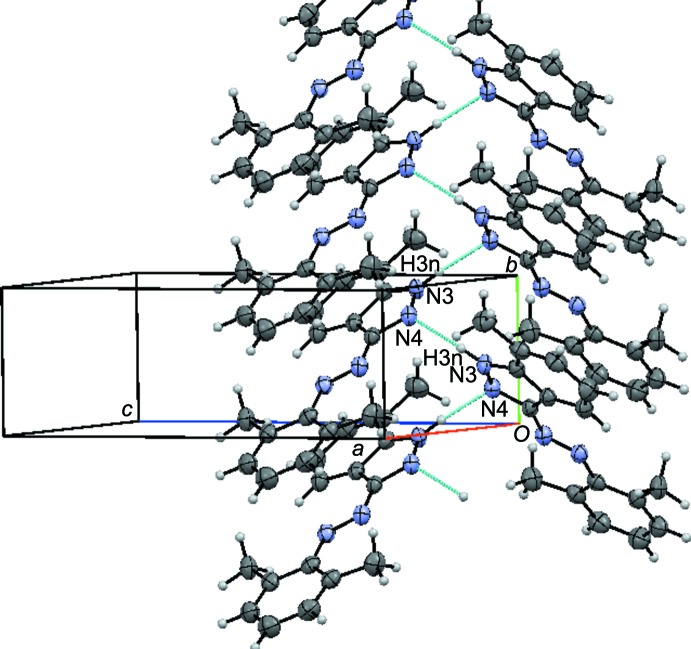
A view of the N—H⋯N hydrogen bonds (blue dashed lines) present in the crystal lattice of the title compound.

In the crystal, mol­ecules are helically connected along the *b*-axis direction by N—H⋯N hydrogen bonds (Table 1 ▸ and Fig. 2 ▸). As a result, chiral crystals of achiral mol­ecules are generated. The angles between the planes of neighbouring mol­ecules in the hydrogen-bonded chains is 82.6 (2)°. Many examples of such achiral mol­ecules forming chiral crystals have been reported, but the prediction of chiral crystallization is still not possible (Koshima & Matsuura, 1998 ▸; Matsuura & Koshima, 2005 ▸).

**Figure 3:**
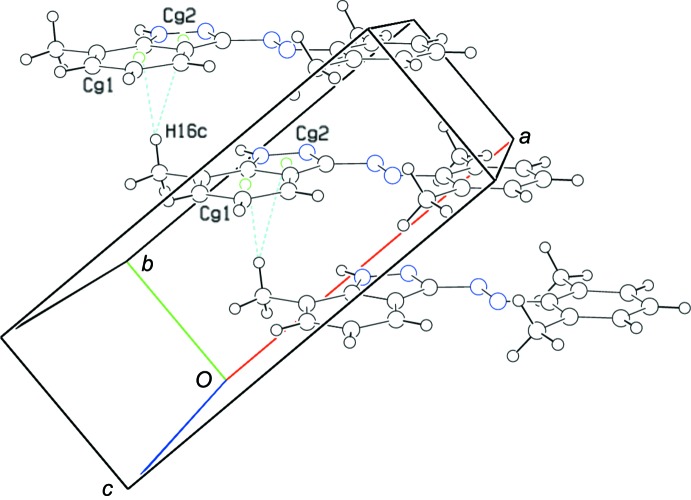
A view of the various C—H⋯π inter­actions (blue dashed lines) present in the crystal lattice of the title compound.

In addition, weak supra­molecular inter­actions, such as the C16—H16*c*⋯*Cg*1 (2.844 Å) and C16—H16*c*⋯*Cg*3 (2.929 Å) C—H⋯π hydrogen bonds, are also found (Table 1 ▸ and Fig. 3 ▸).

## Database survey   

A similar compound, *i.e.* 7-methyl-1*H*-indazole (CCDC refcode 263698; Foces-Foces, 2005 ▸), has already been reported and shows a structure comparable with that of the title compound. However, surveys of the Cambridge Structural Database (CSD, Version 5.38; Groom *et al.*, 2016 ▸) for the title compound revealed no hits. To our knowledge, this is the first crystal structure reported for indazole-type azo dyes.

## Synthesis and crystallization   

A mixture of 2,6-di­methyl­aniline (0.4847 g, 4.000 mmol), concentrated hydro­chloric acid (37%, 1 ml) and water was heated and completely dissolved. The mixture was cooled in an ice bath and NaNO_2_ (0.2967 g, 4.300 mmol) in 4.5 ml water was added. The reaction mixture was stirred at 273 K for 30 min and then salicyl­aldehyde (0.4885 g, 4.000 mmol) in 10 ml of a 10% NaOH aqueous solution was added dropwise and allowed to stir for an additional 1 h. The obtained orange precipitate was filtered off, washed with water and dried in a desiccator for several days (yield 0.2650 g, 26.06%). This crude orange compound was recrystallized by slow evaporation from acetone to give orange prismatic single crystals. IR (KBr, cm^−1^): 746 (*s*), 1147 (*s*), 1162 (*s*), 1425 (*m*), 2923 (*s*), 3136 (*br*). ^1^H NMR (300 MHz, DMSO): δ 2.36 (*s*, 6H), 2.56 (*s*, 3H), 7.18–7.23 (*m*, 5H), 8.02 (*d*, 1H)

## Refinement   

Crystal data, data collection and structure refinement details are summarized in Table 2 ▸. All H atoms were located in difference Fourier maps. C-bound H atoms were constrained using a riding model [C—H = 0.95 Å and *U*_iso_(H) = 1.2*U*_eq_(C) for aromatic H atoms, and C—H = 0.98 Å and *U*_iso_(H) = 1.5*U*_eq_(C) for methyl H atoms]. N-bound H atoms were constrained using a riding model [N—H = 0.88 Å and *U*_iso_(H) = 1.2*U*_eq_(N)].

## Supplementary Material

Crystal structure: contains datablock(s) global. DOI: 10.1107/S2056989018012483/eb2011sup1.cif

CCDC reference: 1865810

Additional supporting information:  crystallographic information; 3D view; checkCIF report

## Figures and Tables

**Table 1:** Hydrogen-bond geometry (Å, °) *Cg*1 and *Cg*2 are the centroids of the C9–C14 and C9–C10/C15/N3–N4 rings, respectively.

*D*—H⋯*A*	*D*—H	H⋯*A*	*D*⋯*A*	*D*—H⋯*A*
N3—H3*n*⋯N4^i^	0.88	2.06	2.851 (5)	149
C16^ii^—H16*c* ^ii^⋯*Cg*1	0.98	2.84	3.544	129
C16^ii^—H16*c* ^ii^⋯*Cg*3	0.98	2.93	3.832	154

Symmetry codes: (i) −*x* + 1, *y* + 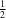, −*z*; (ii) *x*, *y* − 1, *z*.

**Table 2:** Experimental details

Crystal data	
Chemical formula	C_16_H_16_N_4_
*M*_r_	264.33
Crystal system, space group	Monoclinic, *P*2_1_
Temperature (K)	173
*a*, *b*, *c* (Å)	11.052 (8), 4.565 (4), 13.541 (10)
β (°)	97.997 (11)
*V* (Å^3^)	676.5 (9)
*Z*	2
Radiation type	Mo *K*α
μ (mm^−1^)	0.08
Crystal size (mm)	0.30 × 0.12 × 0.09

Data collection	
Diffractometer	Bruker APEXII CCD
Absorption correction	Multi-scan (*SADABS*; Bruker, 2001 ▸)
*T*_min_, *T*_max_	0.323, 0.746
No. of measured, independent and observed [*I* > 2σ(*I*)] reflections	3767, 2682, 2055
*R*_int_	0.053
(sin θ/λ)_max_ (Å^−1^)	0.655

Refinement	
*R*[*F* ^2^ > 2σ(*F* ^2^)], *wR*(*F* ^2^), *S*	0.067, 0.180, 1.01
No. of reflections	2682
No. of parameters	184
No. of restraints	1
H-atom treatment	H-atom parameters constrained
Δρ_max_, Δρ_min_ (e Å^−3^)	0.28, −0.34
Absolute structure	Flack *x* determined using 636 quotients [(*I* ^+^)−(*I* ^−^)]/[(*I* ^+^)+(*I* ^−^)] (Parsons *et al.*, 2013 ▸
Absolute structure parameter	−10.0 (10)

Computer programs: *APEX2* and *SAINT* (Bruker, 2014 ▸), *SHELXT* (Sheldrick, 2015*a* ▸), *SHELXL2014* (Sheldrick, 2015*b* ▸), *ORTEP-3 for Windows* (Farrugia, 2012 ▸), *Mercury* (Macrae *et al.*, 2006 ▸), *SHELXTL* (Sheldrick, 2008 ▸) and *publCIF* (Westrip, 2010 ▸).

## supplementary crystallographic information

### Crystal data

**Table:**

C_16_H_16_N_4_	*F*(000) = 280
*M**_r_* = 264.33	*D*_x_ = 1.297 Mg m^−^^3^
Monoclinic, *P*2_1_	Mo *K*α radiation, λ = 0.71073 Å
*a* = 11.052 (8) Å	Cell parameters from 1267 reflections
*b* = 4.565 (4) Å	θ = 2.6–26.0°
*c* = 13.541 (10) Å	µ = 0.08 mm^−^^1^
β = 97.997 (11)°	*T* = 173 K
*V* = 676.5 (9) Å^3^	Prism, orange
*Z* = 2	0.30 × 0.12 × 0.09 mm

### Data collection

**Table:**

Bruker APEXII CCD diffractometer	2682 independent reflections
Radiation source: fine-focus sealed tube	2055 reflections with *I* > 2σ(*I*)
Detector resolution: 8.3333 pixels mm^-1^	*R*_int_ = 0.053
φ and ω scans	θ_max_ = 27.7°, θ_min_ = 1.5°
Absorption correction: multi-scan (SADABS; Bruker, 2001)	*h* = −11→14
*T*_min_ = 0.323, *T*_max_ = 0.746	*k* = −5→5
3767 measured reflections	*l* = −17→15

### Refinement

**Table:**

Refinement on *F*^2^	Secondary atom site location: difference Fourier map
Least-squares matrix: full	Hydrogen site location: inferred from neighbouring sites
*R*[*F*^2^ > 2σ(*F*^2^)] = 0.067	H-atom parameters constrained
*wR*(*F*^2^) = 0.180	*w* = 1/[σ^2^(*F*_o_^2^) + (0.0993*P*)^2^] where *P* = (*F*_o_^2^ + 2*F*_c_^2^)/3
*S* = 1.01	(Δ/σ)_max_ < 0.001
2682 reflections	Δρ_max_ = 0.28 e Å^−^^3^
184 parameters	Δρ_min_ = −0.34 e Å^−^^3^
1 restraint	Absolute structure: Flack *x* determined using 636 quotients [(I+)-(I-)]/[(I+)+(I-)] (Parsons *et al.*, 2013
Primary atom site location: structure-invariant direct methods	Absolute structure parameter: −10.0 (10)

### Special details

**Table:**

Geometry. All esds (except the esd in the dihedral angle between two l.s. planes) are estimated using the full covariance matrix. The cell esds are taken into account individually in the estimation of esds in distances, angles and torsion angles; correlations between esds in cell parameters are only used when they are defined by crystal symmetry. An approximate (isotropic) treatment of cell esds is used for estimating esds involving l.s. planes.

### Fractional atomic coordinates and isotropic or equivalent isotropic displacement parameters (Å^2^)

**Table:**

	*x*	*y*	*z*	*U*_iso_*/*U*_eq_	
N3	0.4743 (3)	0.9544 (8)	0.0970 (2)	0.0333 (8)	
H3n	0.4345	1.067	0.0507	0.04*	
N1	0.7288 (3)	0.3286 (8)	0.2528 (2)	0.0334 (8)	
N4	0.5704 (3)	0.7839 (8)	0.0832 (2)	0.0334 (8)	
N2	0.7017 (3)	0.4534 (8)	0.1693 (2)	0.0342 (8)	
C10	0.5295 (3)	0.7301 (9)	0.2424 (3)	0.0302 (9)	
C15	0.6037 (3)	0.6474 (9)	0.1687 (3)	0.0298 (8)	
C9	0.4468 (3)	0.9302 (9)	0.1914 (3)	0.0318 (9)	
C1	0.8281 (3)	0.1267 (9)	0.2607 (3)	0.0327 (9)	
C14	0.3515 (3)	1.0650 (10)	0.2343 (3)	0.0371 (10)	
C11	0.5210 (3)	0.6599 (10)	0.3412 (3)	0.0374 (10)	
H11	0.5759	0.5253	0.3774	0.045*	
C6	0.8553 (3)	0.0120 (9)	0.3576 (3)	0.0359 (10)	
C2	0.8945 (3)	0.0392 (10)	0.1838 (3)	0.0374 (10)	
C5	0.9517 (4)	−0.1836 (10)	0.3778 (3)	0.0432 (10)	
H5	0.9722	−0.2586	0.4434	0.052*	
C13	0.3463 (4)	0.9899 (10)	0.3307 (3)	0.0423 (11)	
H13	0.2839	1.073	0.3634	0.051*	
C3	0.9897 (4)	−0.1594 (11)	0.2094 (4)	0.0450 (11)	
H3	1.0367	−0.2205	0.1595	0.054*	
C8	0.7839 (3)	0.0999 (11)	0.4390 (3)	0.0423 (11)	
H8a	0.8096	−0.0192	0.4984	0.063*	
H8b	0.6966	0.0693	0.4167	0.063*	
H8c	0.7988	0.3073	0.455	0.063*	
C4	1.0178 (4)	−0.2699 (11)	0.3043 (4)	0.0503 (12)	
H4	1.0829	−0.406	0.3189	0.06*	
C12	0.4300 (4)	0.7932 (11)	0.3843 (3)	0.0470 (12)	
H12	0.423	0.7523	0.4521	0.056*	
C7	0.8686 (4)	0.1420 (12)	0.0775 (3)	0.0467 (11)	
H7a	0.8869	0.3516	0.0744	0.07*	
H7b	0.7823	0.1085	0.0522	0.07*	
H7c	0.9198	0.0329	0.0367	0.07*	
C16	0.2633 (4)	1.2688 (12)	0.1738 (4)	0.0497 (12)	
H16a	0.1983	1.3235	0.2127	0.075*	
H16b	0.2275	1.1704	0.1123	0.075*	
H16c	0.3067	1.4453	0.1571	0.075*	

### Atomic displacement parameters (Å^2^)

**Table:**

	*U*^11^	*U*^22^	*U*^33^	*U*^12^	*U*^13^	*U*^23^
N3	0.0304 (16)	0.0403 (19)	0.0275 (16)	0.0049 (15)	−0.0023 (12)	0.0025 (15)
N1	0.0317 (16)	0.036 (2)	0.0316 (17)	−0.0040 (15)	0.0026 (12)	0.0015 (16)
N4	0.0327 (16)	0.0404 (19)	0.0264 (16)	−0.0015 (15)	0.0014 (11)	0.0010 (15)
N2	0.0321 (16)	0.041 (2)	0.0294 (17)	−0.0021 (15)	0.0036 (12)	−0.0001 (16)
C10	0.0258 (17)	0.033 (2)	0.0308 (19)	−0.0040 (15)	0.0022 (13)	−0.0006 (16)
C15	0.0321 (18)	0.033 (2)	0.0246 (18)	−0.0035 (17)	0.0039 (13)	−0.0007 (16)
C9	0.0298 (18)	0.033 (2)	0.032 (2)	−0.0044 (16)	0.0030 (14)	−0.0029 (18)
C1	0.0253 (18)	0.033 (2)	0.040 (2)	−0.0062 (17)	0.0044 (14)	−0.0061 (19)
C14	0.0299 (19)	0.036 (2)	0.046 (2)	−0.0031 (17)	0.0067 (16)	−0.0062 (19)
C11	0.038 (2)	0.044 (3)	0.030 (2)	−0.0034 (19)	0.0046 (15)	0.0007 (19)
C6	0.0288 (19)	0.038 (2)	0.040 (2)	−0.0060 (18)	0.0002 (15)	−0.0015 (19)
C2	0.033 (2)	0.039 (2)	0.041 (2)	−0.0055 (18)	0.0074 (16)	−0.0062 (19)
C5	0.034 (2)	0.045 (3)	0.049 (3)	0.002 (2)	−0.0040 (17)	−0.002 (2)
C13	0.037 (2)	0.045 (3)	0.047 (2)	0.002 (2)	0.0119 (18)	−0.010 (2)
C3	0.033 (2)	0.048 (3)	0.055 (3)	0.0017 (19)	0.0086 (18)	−0.010 (2)
C8	0.034 (2)	0.057 (3)	0.034 (2)	−0.002 (2)	−0.0004 (15)	0.002 (2)
C4	0.036 (2)	0.051 (3)	0.063 (3)	0.006 (2)	0.0011 (19)	−0.002 (2)
C12	0.054 (3)	0.056 (3)	0.034 (2)	−0.002 (2)	0.0164 (19)	−0.002 (2)
C7	0.043 (2)	0.059 (3)	0.040 (2)	0.002 (2)	0.0139 (17)	−0.005 (2)
C16	0.039 (2)	0.047 (3)	0.063 (3)	0.007 (2)	0.0066 (19)	0.002 (3)

### Geometric parameters (Å, º)

**Table:**

N3—N4	1.351 (4)	C2—C3	1.396 (6)
N3—C9	1.359 (5)	C2—C7	1.503 (6)
N3—H3n	0.88	C5—C4	1.371 (6)
N1—N2	1.265 (4)	C5—H5	0.95
N1—C1	1.426 (5)	C13—C12	1.415 (6)
N4—C15	1.322 (5)	C13—H13	0.95
N2—C15	1.399 (5)	C3—C4	1.375 (7)
C10—C11	1.391 (5)	C3—H3	0.95
C10—C9	1.404 (6)	C8—H8a	0.98
C10—C15	1.428 (5)	C8—H8b	0.98
C9—C14	1.412 (5)	C8—H8c	0.98
C1—C6	1.406 (6)	C4—H4	0.95
C1—C2	1.413 (5)	C12—H12	0.95
C14—C13	1.358 (6)	C7—H7a	0.98
C14—C16	1.505 (6)	C7—H7b	0.98
C11—C12	1.374 (6)	C7—H7c	0.98
C11—H11	0.95	C16—H16a	0.98
C6—C5	1.388 (5)	C16—H16b	0.98
C6—C8	1.496 (6)	C16—H16c	0.98
			
N4—N3—C9	111.5 (3)	C6—C5—H5	119.5
N4—N3—H3n	124.2	C14—C13—C12	122.8 (4)
C9—N3—H3n	124.2	C14—C13—H13	118.6
N2—N1—C1	116.2 (3)	C12—C13—H13	118.6
C15—N4—N3	106.2 (3)	C4—C3—C2	122.4 (4)
N1—N2—C15	112.1 (3)	C4—C3—H3	118.8
C11—C10—C9	119.8 (4)	C2—C3—H3	118.8
C11—C10—C15	137.1 (4)	C6—C8—H8a	109.5
C9—C10—C15	103.1 (3)	C6—C8—H8b	109.5
N4—C15—N2	115.1 (3)	H8a—C8—H8b	109.5
N4—C15—C10	111.8 (3)	C6—C8—H8c	109.5
N2—C15—C10	133.1 (4)	H8a—C8—H8c	109.5
N3—C9—C10	107.5 (3)	H8b—C8—H8c	109.5
N3—C9—C14	129.0 (4)	C5—C4—C3	119.9 (4)
C10—C9—C14	123.5 (4)	C5—C4—H4	120.0
C6—C1—C2	121.1 (4)	C3—C4—H4	120.0
C6—C1—N1	111.9 (3)	C11—C12—C13	121.8 (4)
C2—C1—N1	127.0 (4)	C11—C12—H12	119.1
C13—C14—C9	114.8 (4)	C13—C12—H12	119.1
C13—C14—C16	124.6 (4)	C2—C7—H7a	109.5
C9—C14—C16	120.6 (4)	C2—C7—H7b	109.5
C12—C11—C10	117.2 (4)	H7a—C7—H7b	109.5
C12—C11—H11	121.4	C2—C7—H7c	109.5
C10—C11—H11	121.4	H7a—C7—H7c	109.5
C5—C6—C1	118.8 (4)	H7b—C7—H7c	109.5
C5—C6—C8	119.8 (4)	C14—C16—H16a	109.5
C1—C6—C8	121.4 (4)	C14—C16—H16b	109.5
C3—C2—C1	116.8 (4)	H16a—C16—H16b	109.5
C3—C2—C7	118.5 (3)	C14—C16—H16c	109.5
C1—C2—C7	124.6 (4)	H16a—C16—H16c	109.5
C4—C5—C6	120.9 (4)	H16b—C16—H16c	109.5
C4—C5—H5	119.5		
			
C9—N3—N4—C15	0.7 (4)	C10—C9—C14—C16	−177.7 (4)
C1—N1—N2—C15	−179.8 (3)	C9—C10—C11—C12	0.1 (6)
N3—N4—C15—N2	178.8 (3)	C15—C10—C11—C12	−178.5 (5)
N3—N4—C15—C10	−0.7 (4)	C2—C1—C6—C5	2.0 (6)
N1—N2—C15—N4	−179.6 (3)	N1—C1—C6—C5	−178.7 (3)
N1—N2—C15—C10	−0.2 (6)	C2—C1—C6—C8	−178.9 (4)
C11—C10—C15—N4	179.2 (4)	N1—C1—C6—C8	0.4 (5)
C9—C10—C15—N4	0.5 (4)	C6—C1—C2—C3	−1.6 (6)
C11—C10—C15—N2	−0.2 (8)	N1—C1—C2—C3	179.2 (3)
C9—C10—C15—N2	−178.9 (4)	C6—C1—C2—C7	177.6 (4)
N4—N3—C9—C10	−0.4 (4)	N1—C1—C2—C7	−1.6 (7)
N4—N3—C9—C14	−178.1 (4)	C1—C6—C5—C4	−1.6 (6)
C11—C10—C9—N3	−179.0 (4)	C8—C6—C5—C4	179.3 (4)
C15—C10—C9—N3	0.0 (4)	C9—C14—C13—C12	0.3 (6)
C11—C10—C9—C14	−1.2 (6)	C16—C14—C13—C12	178.9 (4)
C15—C10—C9—C14	177.8 (4)	C1—C2—C3—C4	0.8 (7)
N2—N1—C1—C6	176.8 (3)	C7—C2—C3—C4	−178.5 (4)
N2—N1—C1—C2	−3.9 (6)	C6—C5—C4—C3	0.8 (7)
N3—C9—C14—C13	178.3 (4)	C2—C3—C4—C5	−0.5 (7)
C10—C9—C14—C13	1.0 (6)	C10—C11—C12—C13	1.2 (6)
N3—C9—C14—C16	−0.3 (7)	C14—C13—C12—C11	−1.4 (7)

### Hydrogen-bond geometry (Å, º)

**Table:**

*D*—H···*A*	*D*—H	H···*A*	*D*···*A*	*D*—H···*A*
N3—H3*n*···N4^i^	0.88	2.06	2.851 (5)	149
C16^ii^—H16*c*^ii^···*Cg*1	0.98	2.84	3.544	129
C16^ii^—H16*c*^ii^···*Cg*2	0.98	2.93	3.832	154

Symmetry codes: (i) −*x*+1, *y*+1/2, −*z*; (ii) *x*, *y*−1, *z*.
